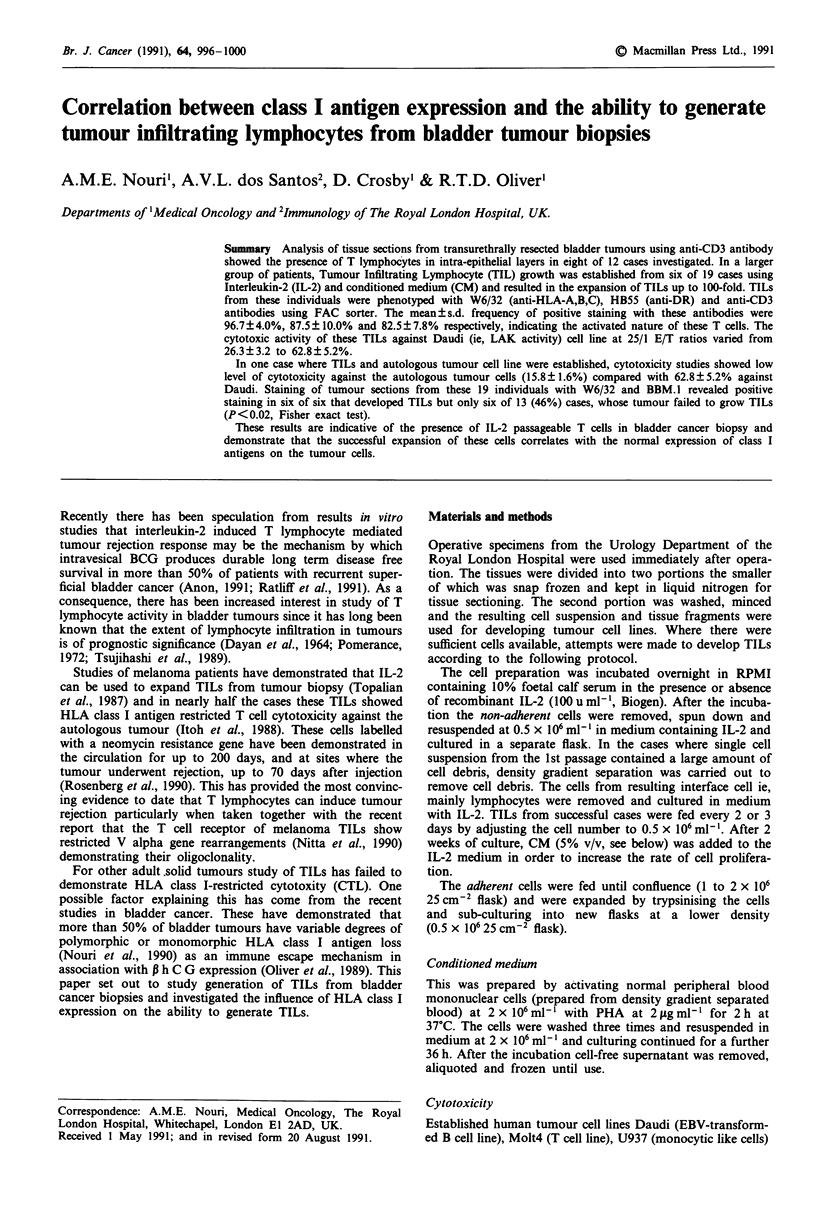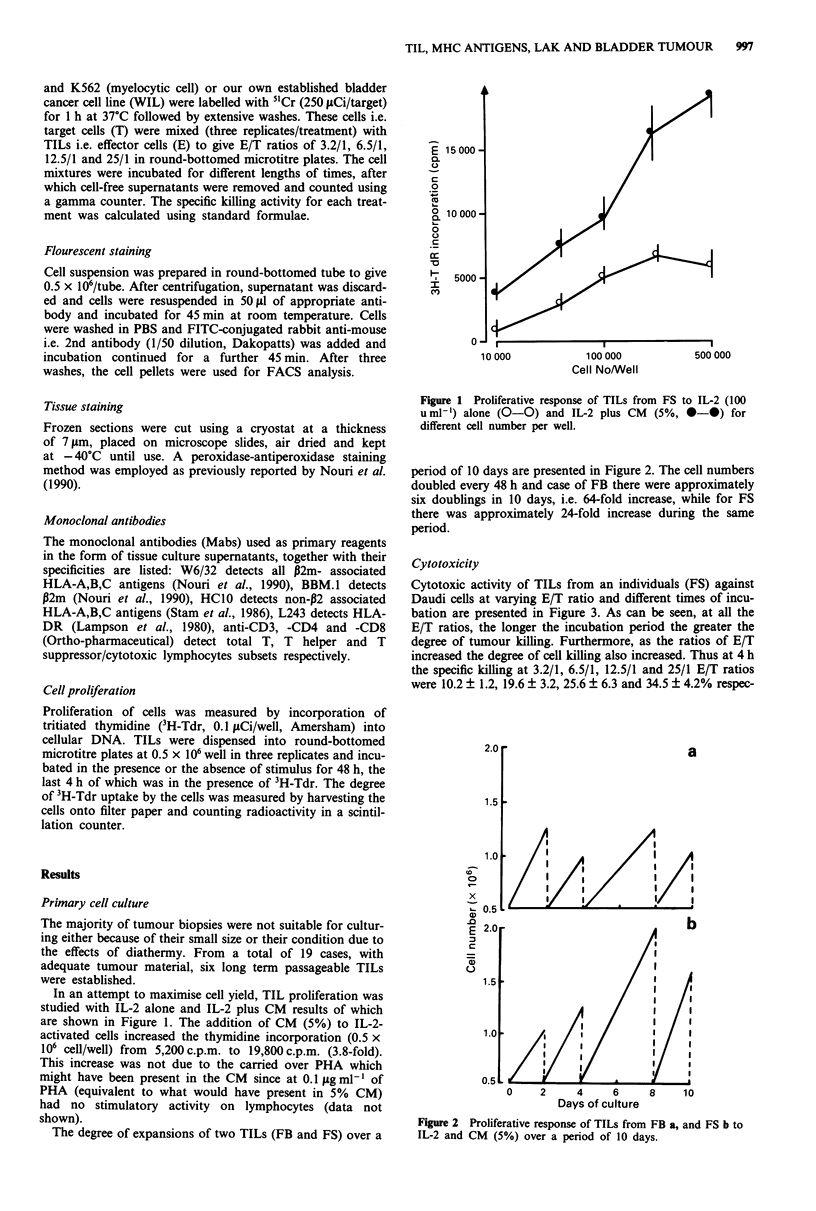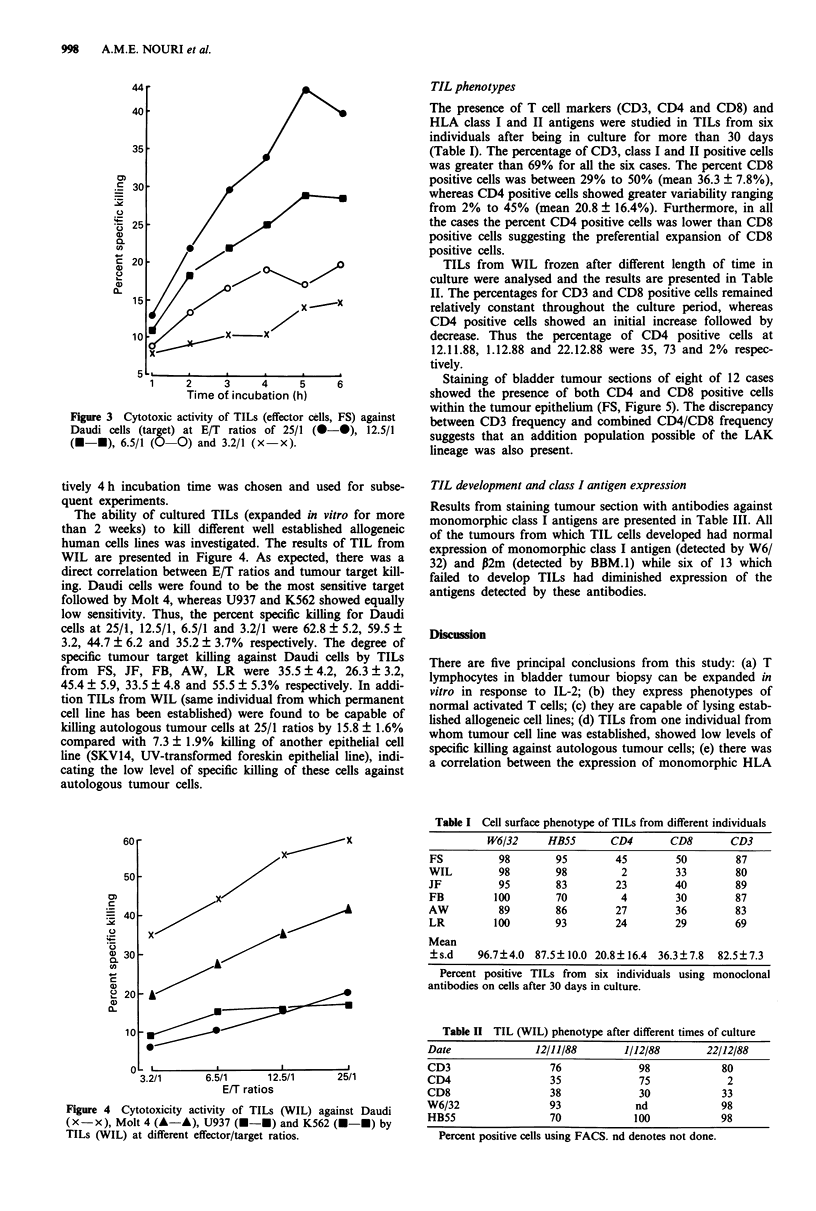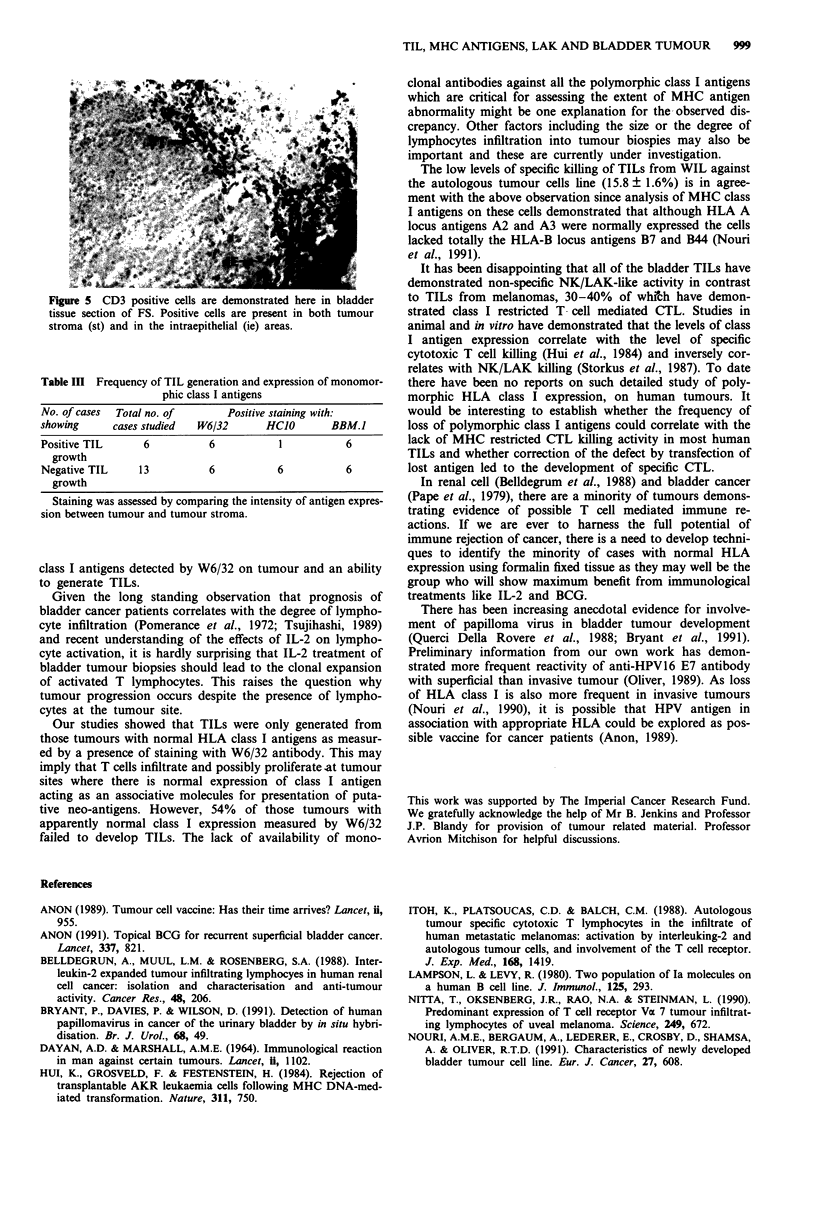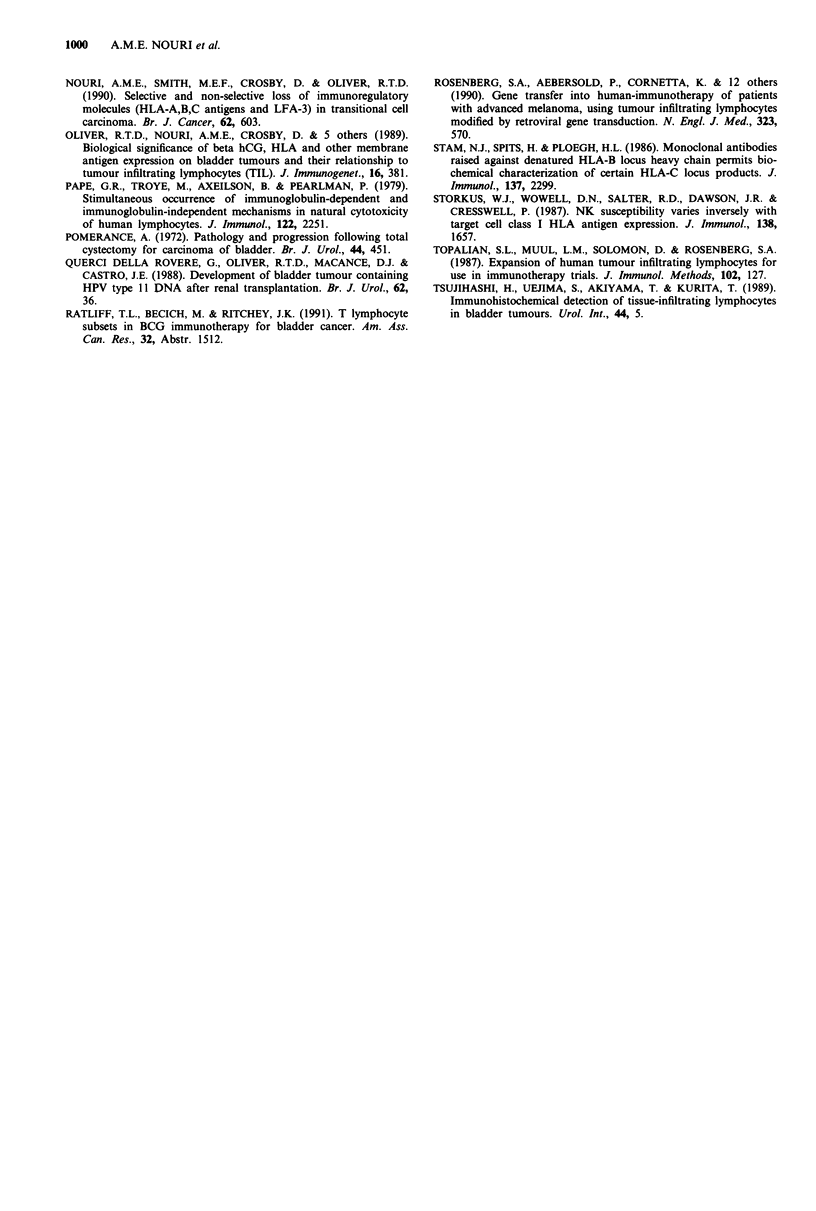# Correlation between class I antigen expression and the ability to generate tumour infiltrating lymphocytes from bladder tumour biopsies.

**DOI:** 10.1038/bjc.1991.454

**Published:** 1991-12

**Authors:** A. M. Nouri, A. V. dos Santos, D. Crosby, R. T. Oliver

**Affiliations:** Department of Medical Oncology, Royal London Hospital, UK.

## Abstract

**Images:**


Br. J. Cancer (1991), 64, 996-1000 © Macmillan Press Ltd., 1991

### Correlation between class I antigen expression and the ability to generate tumour infiltrating lymphocytes from bladder tumour biopsies

A.M.E. Nouri[1], A.V.L. dos Santos[2], D. Crosby[1] & R.T.D. Oliver[1]

*Departments of [1]Medical Oncology and [2]Immunology of The Royal London Hospital, UK.*

**Summary** Analysis of tissue sections from transurethrally resected bladder tumours using anti-CD3 antibody showed the presence of T lymphocytes in intra-epithelial layers in eight of 12 cases investigated. In a larger group of patients, Tumour Infiltrating Lymphocyte (TIL) growth was established from six of 19 cases using Interleukin-2 (IL-2) and conditioned medium (CM) and resulted in the expansion of TILs up to 100-fold. TILs from these individuals were phenotyped with W6/32 (anti-HLA-A,B,C), HB55 (anti-DR) and anti-CD3 antibodies using FAC sorter. The mean±s.d. frequency of positive staining with these antibodies were 96.7±4.0%, 87.5±10.0% and 82.5±7.8% respectively, indicating the activated nature of these T cells. The cytotoxic activity of these TILs against Daudi (ie, LAK activity) cell line at 25/1 E/T ratios varied from 26.3±3.2 to 62.8±5.2%.

In one case where TILs and autologous tumour cell line were established, cytotoxicity studies showed low level of cytotoxicity against the autologous tumour cells (15.8±1.6%) compared with 62.8±5.2% against Daudi. Staining of tumour sections from these 19 individuals with W6/32 and BBM.1 revealed positive staining in six of six that developed TILs but only six of 13 (46%) cases, whose tumour failed to grow TILs ($P<0.02$, Fisher exact test).

These results are indicative of the presence of IL-2 passageable T cells in bladder cancer biopsy and demonstrate that the successful expansion of these cells correlates with the normal expression of class I antigens on the tumour cells.

Recently there has been speculation from results *in vitro* studies that interleukin-2 induced T lymphocyte mediated tumour rejection response may be the mechanism by which intravesical BCG produces durable long term disease free survival in more than 50% of patients with recurrent superficial bladder cancer (Anon, 1991; Ratliff *et al.*, 1991). As a consequence, there has been increased interest in study of T lymphocyte activity in bladder tumours since it has long been known that the extent of lymphocyte infiltration in tumours is of prognostic significance (Dayan *et al.*, 1964; Pomerance, 1972; Tsujihashi *et al.*, 1989).

Studies of melanoma patients have demonstrated that IL-2 can be used to expand TILs from tumour biopsy (Topalian *et al.*, 1987) and in nearly half the cases these TILs showed HLA class I antigen restricted T cell cytotoxicity against the autologous tumour (Itoh *et al.*, 1988). These cells labelled with a neomycin resistance gene have been demonstrated in the circulation for up to 200 days, and at sites where the tumour underwent rejection, up to 70 days after injection (Rosenberg *et al.*, 1990). This has provided the most convincing evidence to date that T lymphocytes can induce tumour rejection particularly when taken together with the recent report that the T cell receptor of melanoma TILs show restricted V alpha gene rearrangements (Nitta *et al.*, 1990) demonstrating their oligoclonality.

For other adult solid tumours study of TILs has failed to demonstrate HLA class I-restricted cytotoxity (CTL). One possible factor explaining this has come from the recent studies in bladder cancer. These have demonstrated that more than 50% of bladder tumours have variable degrees of polymorphic or monomorphic HLA class I antigen loss (Nouri *et al.*, 1990) as an immune escape mechanism in association with $\beta$ h C G expression (Oliver *et al.*, 1989). This paper set out to study generation of TILs from bladder cancer biopsies and investigated the influence of HLA class I expression on the ability to generate TILs.

Correspondence: A.M.E. Nouri, Medical Oncology, The Royal London Hospital, Whitechapel, London E1 2AD, UK.
Received 1 May 1991; and in revised form 20 August 1991.

### Materials and methods

Operative specimens from the Urology Department of the Royal London Hospital were used immediately after operation. The tissues were divided into two portions the smaller of which was snap frozen and kept in liquid nitrogen for tissue sectioning. The second portion was washed, minced and the resulting cell suspension and tissue fragments were used for developing tumour cell lines. Where there were sufficient cells available, attempts were made to develop TILs according to the following protocol.

The cell preparation was incubated overnight in RPMI containing 10% foetal calf serum in the presence or absence of recombinant IL-2 (100 u ml$^{-1}$, Biogen). After the incubation the *non-adherent* cells were removed, spun down and resuspended at $0.5 \times 10^6$ ml$^{-1}$ in medium containing IL-2 and cultured in a separate flask. In the cases where single cell suspension from the 1st passage contained a large amount of cell debris, density gradient separation was carried out to remove cell debris. The cells from resulting interface cell ie, mainly lymphocytes were removed and cultured in medium with IL-2. TILs from successful cases were fed every 2 or 3 days by adjusting the cell number to $0.5 \times 10^6$ ml$^{-1}$. After 2 weeks of culture, CM (5% v/v, see below) was added to the IL-2 medium in order to increase the rate of cell proliferation.

The *adherent* cells were fed until confluence (1 to $2 \times 10^6$ 25 cm$^{-2}$ flask) and were expanded by trypsinising the cells and sub-culturing into new flasks at a lower density ($0.5 \times 10^6$ 25 cm$^{-2}$ flask).

### *Conditioned medium*

This was prepared by activating normal peripheral blood mononuclear cells (prepared from density gradient separated blood) at $2 \times 10^6$ ml$^{-1}$ with PHA at 2 $\mu$g ml$^{-1}$ for 2 h at 37°C. The cells were washed three times and resuspended in medium at $2 \times 10^6$ ml$^{-1}$ and culturing continued for a further 36 h. After the incubation cell-free supernatant was removed, aliquoted and frozen until use.

### *Cytotoxicity*

Established human tumour cell lines Daudi (EBV-transformed B cell line), Molt4 (T cell line), U937 (monocytic like cells)

and K562 (myelocytic cell) or our own established bladder cancer cell line (WIL) were labelled with $^{51}Cr$ (250 μCi/target) for 1 h at 37°C followed by extensive washes. These cells i.e. target cells (T) were mixed (three replicates/treatment) with TILs i.e. effector cells (E) to give E/T ratios of 3.2/1, 6.5/1, 12.5/1 and 25/1 in round-bottomed microtitre plates. The cell mixtures were incubated for different lengths of times, after which cell-free supernatants were removed and counted using a gamma counter. The specific killing activity for each treatment was calculated using standard formulae.

*Flourescent staining*

Cell suspension was prepared in round-bottomed tube to give $0.5 \times 10^6$/tube. After centrifugation, supernatant was discarded and cells were resuspended in 50 μl of appropriate antibody and incubated for 45 min at room temperature. Cells were washed in PBS and FITC-conjugated rabbit anti-mouse i.e. 2nd antibody (1/50 dilution, Dakopatts) was added and incubation continued for a further 45 min. After three washes, the cell pellets were used for FACS analysis.

*Tissue staining*

Frozen sections were cut using a cryostat at a thickness of 7 μm, placed on microscope slides, air dried and kept at −40°C until use. A peroxidase-antiperoxidase staining method was employed as previously reported by Nouri *et al.* (1990).

*Monoclonal antibodies*

The monoclonal antibodies (Mabs) used as primary reagents in the form of tissue culture supernatants, together with their specificities are listed: W6/32 detects all β2m- associated HLA-A,B,C antigens (Nouri *et al.*, 1990), BBM.1 detects β2m (Nouri *et al.*, 1990), HC10 detects non-β2 associated HLA-A,B,C antigens (Stam *et al.*, 1986), L243 detects HLA-DR (Lampson *et al.*, 1980), anti-CD3, -CD4 and -CD8 (Ortho-pharmaceutical) detect total T, T helper and T suppressor/cytotoxic lymphocytes subsets respectively.

*Cell proliferation*

Proliferation of cells was measured by incorporation of tritiated thymidine ($^3$H-Tdr, 0.1 μCi/well, Amersham) into cellular DNA. TILs were dispensed into round-bottomed microtitre plates at $0.5 \times 10^6$ well in three replicates and incubated in the presence or the absence of stimulus for 48 h, the last 4 h of which was in the presence of $^3$H-Tdr. The degree of $^3$H-Tdr uptake by the cells was measured by harvesting the cells onto filter paper and counting radioactivity in a scintillation counter.

### Results

*Primary cell culture*

The majority of tumour biopsies were not suitable for culturing either because of their small size or their condition due to the effects of diathermy. From a total of 19 cases, with adequate tumour material, six long term passageable TILs were established.

In an attempt to maximise cell yield, TIL proliferation was studied with IL-2 alone and IL-2 plus CM results of which are shown in Figure 1. The addition of CM (5%) to IL-2-activated cells increased the thymidine incorporation ($0.5 \times 10^6$ cell/well) from 5,200 c.p.m. to 19,800 c.p.m. (3.8-fold). This increase was not due to the carried over PHA which might have been present in the CM since at 0.1 μg ml$^{-1}$ of PHA (equivalent to what would have present in 5% CM) had no stimulatory activity on lymphocytes (data not shown).

The degree of expansions of two TILs (FB and FS) over a

**Figure 1** Proliferative response of TILs from FS to IL-2 (100 u ml$^{-1}$) alone (O—O) and IL-2 plus CM (5%, ●—●) for different cell number per well.

period of 10 days are presented in Figure 2. The cell numbers doubled every 48 h and case of FB there were approximately six doublings in 10 days, i.e. 64-fold increase, while for FS there was approximately 24-fold increase during the same period.

*Cytotoxicity*

Cytotoxic activity of TILs from an individuals (FS) against Daudi cells at varying E/T ratio and different times of incubation are presented in Figure 3. As can be seen, at all the E/T ratios, the longer the incubation period the greater the degree of tumour killing. Furthermore, as the ratios of E/T increased the degree of cell killing also increased. Thus at 4 h the specific killing at 3.2/1, 6.5/1, 12.5/1 and 25/1 E/T ratios were 10.2 ± 1.2, 19.6 ± 3.2, 25.6 ± 6.3 and 34.5 ± 4.2% respec-

**Figure 2** Proliferative response of TILs from FB **a**, and FS **b** to IL-2 and CM (5%) over a period of 10 days.

Figure 3 Cytotoxic activity of TILs (effector cells, FS) against Daudi cells (target) at E/T ratios of 25/1 (●—●), 12.5/1 (■—■), 6.5/1 (O—O) and 3.2/1 (×—×).

tively 4 h incubation time was chosen and used for subsequent experiments.

The ability of cultured TILs (expanded *in vitro* for more than 2 weeks) to kill different well established allogeneic human cells lines was investigated. The results of TIL from WIL are presented in Figure 4. As expected, there was a direct correlation between E/T ratios and tumour target killing. Daudi cells were found to be the most sensitive target followed by Molt 4, whereas U937 and K562 showed equally low sensitivity. Thus, the percent specific killing for Daudi cells at 25/1, 12.5/1, 6.5/1 and 3.2/1 were 62.8 ± 5.2, 59.5 ± 3.2, 44.7 ± 6.2 and 35.2 ± 3.7% respectively. The degree of specific tumour target killing against Daudi cells by TILs from FS, JF, FB, AW, LR were 35.5 ± 4.2, 26.3 ± 3.2, 45.4 ± 5.9, 33.5 ± 4.8 and 55.5 ± 5.3% respectively. In addition TILs from WIL (same individual from which permanent cell line has been established) were found to be capable of killing autologous tumour cells at 25/1 ratios by 15.8 ± 1.6% compared with 7.3 ± 1.9% killing of another epithelial cell line (SKV14, UV-transformed foreskin epithelial line), indicating the low level of specific killing of these cells against autologous tumour cells.

Figure 4 Cytotoxicity activity of TILs (WIL) against Daudi (×—×), Molt 4 (▲—▲), U937 (■—■) and K562 (■—■) by TILs (WIL) at different effector/target ratios.

### *TIL phenotypes*

The presence of T cell markers (CD3, CD4 and CD8) and HLA class I and II antigens were studied in TILs from six individuals after being in culture for more than 30 days (Table I). The percentage of CD3, class I and II positive cells was greater than 69% for all the six cases. The percent CD8 positive cells was between 29% to 50% (mean 36.3 ± 7.8%), whereas CD4 positive cells showed greater variability ranging from 2% to 45% (mean 20.8 ± 16.4%). Furthermore, in all the cases the percent CD4 positive cells was lower than CD8 positive cells suggesting the preferential expansion of CD8 positive cells.

TILs from WIL frozen after different length of time in culture were analysed and the results are presented in Table II. The percentages for CD3 and CD8 positive cells remained relatively constant throughout the culture period, whereas CD4 positive cells showed an initial increase followed by decrease. Thus the percentage of CD4 positive cells at 12.11.88, 1.12.88 and 22.12.88 were 35, 73 and 2% respectively.

Staining of bladder tumour sections of eight of 12 cases showed the presence of both CD4 and CD8 positive cells within the tumour epithelium (FS, Figure 5). The discrepancy between CD3 frequency and combined CD4/CD8 frequency suggests that an addition population possible of the LAK lineage was also present.

### *TIL development and class I antigen expression*

Results from staining tumour section with antibodies against monomorphic class I antigens are presented in Table III. All of the tumours from which TIL cells developed had normal expression of monomorphic class I antigen (detected by W6/32) and β2m (detected by BBM.1) while six of 13 which failed to develop TILs had diminished expression of the antigens detected by these antibodies.

### Discussion

There are five principal conclusions from this study: (a) T lymphocytes in bladder tumour biopsy can be expanded *in vitro* in response to IL-2; (b) they express phenotypes of normal activated T cells; (c) they are capable of lysing established allogeneic cell lines; (d) TILs from one individual from whom tumour cell line was established, showed low levels of specific killing against autologous tumour cells; (e) there was a correlation between the expression of monomorphic HLA

**Table I** Cell surface phenotype of TILs from different individuals

| | *W6/32* | *HB55* | *CD4* | *CD8* | *CD3* |
|---|---|---|---|---|---|
| FS | 98 | 95 | 45 | 50 | 87 |
| WIL | 98 | 98 | 2 | 33 | 80 |
| JF | 95 | 83 | 23 | 40 | 89 |
| FB | 100 | 70 | 4 | 30 | 87 |
| AW | 89 | 86 | 27 | 36 | 83 |
| LR | 100 | 93 | 24 | 29 | 69 |
| Mean ±s.d | 96.7±4.0 | 87.5±10.0 | 20.8±16.4 | 36.3±7.8 | 82.5±7.3 |

Percent positive TILs from six individuals using monoclonal antibodies on cells after 30 days in culture.

**Table II** TIL (WIL) phenotype after different times of culture

| *Date* | *12/11/88* | *1/12/88* | *22/12/88* |
|---|---|---|---|
| CD3 | 76 | 98 | 80 |
| CD4 | 35 | 75 | 2 |
| CD8 | 38 | 30 | 33 |
| W6/32 | 93 | nd | 98 |
| HB55 | 70 | 100 | 98 |

Percent positive cells using FACS. nd denotes not done.

**Figure 5** CD3 positive cells are demonstrated here in bladder tissue section of FS. Positive cells are present in both tumour stroma (st) and in the intraepithelial (ie) areas.

**Table III** Frequency of TIL generation and expression of monomorphic class I antigens

| *No. of cases showing* | *Total no. of cases studied* | *Positive staining with:* W6/32 | HC10 | BBM.1 |
|---|---|---|---|---|
| Positive TIL growth | 6 | 6 | 1 | 6 |
| Negative TIL growth | 13 | 6 | 6 | 6 |

Staining was assessed by comparing the intensity of antigen expression between tumour and tumour stroma.

class I antigens detected by W6/32 on tumour and an ability to generate TILs.

Given the long standing observation that prognosis of bladder cancer patients correlates with the degree of lymphocyte infiltration (Pomerance *et al.*, 1972; Tsujihashi, 1989) and recent understanding of the effects of IL-2 on lymphocyte activation, it is hardly surprising that IL-2 treatment of bladder tumour biopsies should lead to the clonal expansion of activated T lymphocytes. This raises the question why tumour progression occurs despite the presence of lymphocytes at the tumour site.

Our studies showed that TILs were only generated from those tumours with normal HLA class I antigens as measured by a presence of staining with W6/32 antibody. This may imply that T cells infiltrate and possibly proliferate at tumour sites where there is normal expression of class I antigen acting as an associative molecules for presentation of putative neo-antigens. However, 54% of those tumours with apparently normal class I expression measured by W6/32 failed to develop TILs. The lack of availability of monoclonal antibodies against all the polymorphic class I antigens which are critical for assessing the extent of MHC antigen abnormality might be one explanation for the observed discrepancy. Other factors including the size or the degree of lymphocytes infiltration into tumour biospies may also be important and these are currently under investigation.

The low levels of specific killing of TILs from WIL against the autologous tumour cells line (15.8 ± 1.6%) is in agreement with the above observation since analysis of MHC class I antigens on these cells demonstrated that although HLA A locus antigens A2 and A3 were normally expressed the cells lacked totally the HLA-B locus antigens B7 and B44 (Nouri *et al.*, 1991).

It has been disappointing that all of the bladder TILs have demonstrated non-specific NK/LAK-like activity in contrast to TILs from melanomas, 30–40% of which have demonstrated class I restricted T cell mediated CTL. Studies in animal and *in vitro* have demonstrated that the levels of class I antigen expression correlate with the level of specific cytotoxic T cell killing (Hui *et al.*, 1984) and inversely correlates with NK/LAK killing (Storkus *et al.*, 1987). To date there have been no reports on such detailed study of polymorphic HLA class I expression, on human tumours. It would be interesting to establish whether the frequency of loss of polymorphic class I antigens could correlate with the lack of MHC restricted CTL killing activity in most human TILs and whether correction of the defect by transfection of lost antigen led to the development of specific CTL.

In renal cell (Belldegrum *et al.*, 1988) and bladder cancer (Pape *et al.*, 1979), there are a minority of tumours demonstrating evidence of possible T cell mediated immune reactions. If we are ever to harness the full potential of immune rejection of cancer, there is a need to develop techniques to identify the minority of cases with normal HLA expression using formalin fixed tissue as they may well be the group who will show maximum benefit from immunological treatments like IL-2 and BCG.

There has been increasing anecdotal evidence for involvement of papilloma virus in bladder tumour development (Querci Della Rovere *et al.*, 1988; Bryant *et al.*, 1991). Preliminary information from our own work has demonstrated more frequent reactivity of anti-HPV16 E7 antibody with superficial than invasive tumour (Oliver, 1989). As loss of HLA class I is also more frequent in invasive tumours (Nouri *et al.*, 1990), it is possible that HPV antigen in association with appropriate HLA could be explored as possible vaccine for cancer patients (Anon, 1989).

This work was supported by The Imperial Cancer Research Fund. We gratefully acknowledge the help of Mr B. Jenkins and Professor J.P. Blandy for provision of tumour related material. Professor Avrion Mitchison for helpful discussions.

### References

ANON (1989). Tumour cell vaccine: Has their time arrives? *Lancet*, **ii**, 955.

ANON (1991). Topical BCG for recurrent superficial bladder cancer. *Lancet*, **337**, 821.

BELLDEGRUN, A., MUUL, L.M. & ROSENBERG, S.A. (1988). Interleukin-2 expanded tumour infiltrating lymphocyes in human renal cell cancer: isolation and characterisation and anti-tumour activity. *Cancer Res.*, **48**, 206.

BRYANT, P., DAVIES, P. & WILSON, D. (1991). Detection of human papillomavirus in cancer of the urinary bladder by *in situ* hybridisation. *Br. J. Urol.*, **68**, 49.

DAYAN, A.D. & MARSHALL, A.M.E. (1964). Immunological reaction in man against certain tumours. *Lancet*, **ii**, 1102.

HUI, K., GROSVELD, F. & FESTENSTEIN, H. (1984). Rejection of transplantable AKR leukaemia cells following MHC DNA-mediated transformation. *Nature*, **311**, 750.

ITOH, K., PLATSOUCAS, C.D. & BALCH, C.M. (1988). Autologous tumour specific cytotoxic T lymphocytes in the infiltrate of human metastatic melanomas: activation by interleuking-2 and autologous tumour cells, and involvement of the T cell receptor. *J. Exp. Med.*, **168**, 1419.

LAMPSON, L. & LEVY, R. (1980). Two population of Ia molecules on a human B cell line. *J. Immunol.*, **125**, 293.

NITTA, T., OKSENBERG, J.R., RAO, N.A. & STEINMAN, L. (1990). Predominant expression of T cell receptor Vα 7 tumour infiltrating lymphocytes of uveal melanoma. *Science*, **249**, 672.

NOURI, A.M.E., BERGAUM, A., LEDERER, E., CROSBY, D., SHAMSA, A. & OLIVER, R.T.D. (1991). Characteristics of newly developed bladder tumour cell line. *Eur. J. Cancer*, **27**, 608.

NOURI, A.M.E., SMITH, M.E.F., CROSBY, D. & OLIVER, R.T.D. (1990). Selective and non-selective loss of immunoregulatory molecules (HLA-A,B,C antigens and LFA-3) in transitional cell carcinoma. *Br. J. Cancer*, **62**, 603.

OLIVER, R.T.D., NOURI, A.M.E., CROSBY, D. & 5 others (1989). Biological significance of beta hCG, HLA and other membrane antigen expression on bladder tumours and their relationship to tumour infiltrating lymphocytes (TIL). *J. Immunogenet.*, **16**, 381.

PAPE, G.R., TROYE, M., AXEILSON, B. & PEARLMAN, P. (1979). Stimultaneous occurrence of immunoglobulin-dependent and immunoglobulin-independent mechanisms in natural cytotoxicity of human lymphocytes. *J. Immunol.*, **122**, 2251.

POMERANCE, A. (1972). Pathology and progression following total cystectomy for carcinoma of bladder. *Br. J. Urol.*, **44**, 451.

QUERCI DELLA ROVERE, G., OLIVER, R.T.D., MACANCE, D.J. & CASTRO, J.E. (1988). Development of bladder tumour containing HPV type 11 DNA after renal transplantation. *Br. J. Urol.*, **62**, 36.

RATLIFF, T.L., BECICH, M. & RITCHEY, J.K. (1991). T lymphocyte subsets in BCG immunotherapy for bladder cancer. *Am. Ass. Can. Res.*, **32**, Abstr. 1512.

ROSENBERG, S.A., AEBERSOLD, P., CORNETTA, K. & 12 others (1990). Gene transfer into human-immunotherapy of patients with advanced melanoma, using tumour infiltrating lymphocytes modified by retroviral gene transduction. *N. Engl. J. Med.*, **323**, 570.

STAM, N.J., SPITS, H. & PLOEGH, H.L. (1986). Monoclonal antibodies raised against denatured HLA-B locus heavy chain permits biochemical characterization of certain HLA-C locus products. *J. Immunol.*, **137**, 2299.

STORKUS, W.J., WOWELL, D.N., SALTER, R.D., DAWSON, J.R. & CRESSWELL, P. (1987). NK susceptibility varies inversely with target cell class I HLA antigen expression. *J. Immunol.*, **138**, 1657.

TOPALIAN, S.L., MUUL, L.M., SOLOMON, D. & ROSENBERG, S.A. (1987). Expansion of human tumour infiltrating lymphocytes for use in immunotherapy trials. *J. Immunol. Methods*, **102**, 127.

TSUJIHASHI, H., UEJIMA, S., AKIYAMA, T. & KURITA, T. (1989). Immunohistochemical detection of tissue-infiltrating lymphocytes in bladder tumours. *Urol. Int.*, **44**, 5.